# Does potential antibody-dependent enhancement occur during SARS-CoV-2 infection after natural infection or vaccination? A meta-analysis

**DOI:** 10.1186/s12879-022-07735-2

**Published:** 2022-09-19

**Authors:** Lin Gan, Yan Chen, Jinlin Tan, Xuezhi Wang, Dingmei Zhang

**Affiliations:** 1 Department of Epidemiology, School of Public Health, Sun Yat-Sen University, Guangzhou, 510080 Guangdong China; 2 Medical College of Shaoguan University, Shaoguan, 512000 Guangdong China; 3 Foshan No.4 People’s Hospital, Foshan, 528000 Guangdong China

**Keywords:** COVID-19, SARS-CoV-2 vaccine, Vaccination, Reinfection, ADE

## Abstract

Coronavirus disease 2019 (COVID-19) continues to constitute an international public health emergency. Vaccination is a prospective approach to control this pandemic. However, apprehension about the safety of vaccines is a major obstacle to vaccination. Amongst health professionals, one evident concern is the risk of antibody-dependent enhancement (ADE), which may increase the severity of COVID-19. To explore whether ADE occurs in severe acute respiratory syndrome coronavirus 2 (SARS-CoV-2) infections and increase confidence in the safety of vaccination, we conducted a meta-analysis to investigate the relationship between post-immune infection and disease severity from a population perspective. Databases, including PubMed, EMBASE, Chinese National Knowledge Infrastructure, SinoMed, Scopus, Science Direct, and Cochrane Library, were searched for articles on SARS-CoV-2 reinfection published until 25 October 2021. The papers were reviewed for methodological quality, and a random effects model was used to analyse the results. Heterogeneity was assessed using the *I*^2^ statistic. Publication bias was evaluated using a funnel plot and Egger’s test. Eleven studies were included in the final meta-analysis. The pooled results indicated that initial infection and vaccination were protective factors against severe COVID-19 during post-immune infection (*OR* = 0.55, 95%*CI* = 0.31–0.98). A subgroup (post-immune infection after natural infection or vaccination) analysis showed similar results. Primary SARS-CoV-2 infection and vaccination provide adequate protection against severe clinical symptoms after post-immune infection. This finding demonstrates that SARS-CoV-2 may not trigger ADE at the population level.

## Introduction

Severe acute respiratory syndrome coronavirus 2 (SARS-CoV-2), the pathogen that causes coronavirus disease 2019 (COVID-19), has had profound effects on human health globally. As of June 2022, more than 533 million cases of COVID-19 and 6.30 million deaths due to COVID-19 have been reported globally. Patients present with different clinical manifestations and degrees of severity. The majority of COVID-19 patients have a mild form of the disease, and most recover after symptomatic support treatment. However, once severe disease develops, the difficulty of treatment and the risk of death greatly increase. Vaccination is an effective method of preventing infectious diseases. Various types of vaccines, such as inactivated virus, recombinant protein, adenovirus vector, DNA, and mRNA vaccines, have been developed and are expected to provide protection against SARS-CoV-2 infection. However, whether natural infection or vaccination prevents reinfection or breakthrough infection has been a serious concern after the COVID-19 outbreak. It is generally believed that prior infection with SARS-CoV-2 may be protective against reinfection and symptomatic disease. However, the first case of SARS-CoV-2 reinfection was reported in August 2020, and it was rapidly followed by additional cases globally [1–3]. Moreover, with the development of vaccines, another important issue causing concern is antibody-dependent enhancement (ADE), as observed in dengue fever [4]. It has been shown that when patients are infected by one serotype of dengue virus (i.e., primary infection), they produce neutralizing antibodies targeting the same serotype of the virus. However, if they are later infected by another serotype of dengue virus (i.e., secondary infection), the preexisting antibodies cannot fully neutralize the virus. Instead of preventing infection, these antibodies may interact with the virus and complement components and enhance the infection. This phenomenon, known as ADE, usually causes more severe clinical outcomes. It has been documented for other coronaviruses, such as SARS-CoV and Middle East respiratory syndrome-related coronavirus (MERS-CoV) [5, 6]. Several in vitro and in vivo experiments have been conducted to determine whether ADE occurs in COVID-19 patients. One study showed that an inactivated SARS-CoV-2 vaccine elicits strong neutralizing antibodies in mice, rats, and rhesus macaques, with no evidence of ADE [7]. Another study found that antibodies against the SARS-CoV-2 receptor-binding domain and N-terminal domain increase the infection rate in vitro, but do not increase coronavirus replication in mouse or monkey models in vivo [8]. A recent study in Japan reported that the production of enhancing antibodies may be boosted by SARS-CoV-2 infection or vaccination, and these enhancing antibodies may cause ADE [9]. Therefore, no decisive conclusion has been reached regarding whether SARS-CoV-2 infection causes ADE.

The willingness of people to receive SARS-CoV-2 vaccines has been adversely affected by negative news about some of the vaccines. A previous cross-sectional study conducted by our team found that 39.6% of adults in China were unwilling to be or uncertain about being vaccinated against SARS-CoV-2 [10]. Their unwillingness to be vaccinated was due to a fear that the vaccines were not sufficiently safe, or because they planned to observe the effects of vaccination for a certain period before deciding to be vaccinated. Some people were unwilling to be vaccinated because they were apprehensive about the ADE effect of the vaccines. Although large-scale vaccination is required as soon as possible, its implementation has been hindered by the distrust of the public.

To respond to people’s concerns about the safety of vaccines, a meta-analysis was conducted to determine whether patients who experience SARS-CoV-2 reinfection or breakthrough infections have more serious disease manifestations, thus inferring whether the ADE phenomenon occurs in COVID-19 patients. This was achieved by studying the relationship between post-immune infection and disease severity from a population perspective. The articles included in the review covered reinfections after a natural infection and breakthrough infections after vaccination.

## Materials and methods

### Literature search

Articles published up to 25 October 2021 were searched using the databases PubMed, EMBASE, Chinese National Knowledge Infrastructure, SinoMed, Scopus, Science Direct, and Cochrane Library. Databases were searched using Medical Subject Headings terms as follows: (“coronavirus disease 2019” or “COVID-19” or “SARS-CoV-2” or “2019-nCov”) AND (“reinfection” or “re-infection” or “second episode” or “reinfections” or “reactivation” or “vaccination” or “vaccine”) AND (“severe case” or “severe cases” or “severity” or “serious”), without any limitations on language or year of publication.

### Definitions

Post-immune infections refers to reinfection after initial infection, or breakthrough infection after vaccination. Here, infection was defined in accordance with the guidelines of the Centers for Disease Control and Prevention of the United States. The infection criteria included a positive (with a cycle threshold < 33) real-time reverse transcription polymerase chain reaction test at least 45 days after the initial test, accompanied by typical symptoms or epidemiological exposure. Patients infected with SARS-CoV-2 after vaccination were also included in the current study. Severe cases were defined as those with respiratory failure that required mechanical ventilation or shock, or those with other organ failure that necessitated treatment in an intensive care unit. Most of the cases included in the severe case group were consistent with the case determination, while one (Slezak, et al.) of the 11 articles included in this review did not clearly indicate the severity of the disease. We regarded hospitalized patients as having severe disease.

### Inclusion and exclusion criteria

Studies were included in this review if they: (1) investigated breakthrough infection after vaccination or reinfection after an initial infection and (2) reported the number of cases of initial infection and reinfection and the number of severe cases.

Studies were excluded if they: (1) did not report data of the cases or (2) did not report clinical severity data.

In addition, based on the site and date of the study, we determined whether the study groups overlapped, and if so, only the study with the largest population was included.

### Data extraction

Two authors performed the literature search individually and then discussed differences, if any, with a third author. The titles and abstracts of the retrieved articles were screened for eligibility. The following data were extracted from all of the included studies: first author, publication year, country, study type, sample size, sex, age range, and defined clinical outcomes.

### Quality assessment

The Jadad scale and the Newcastle–Ottawa scale (NOS) were used to assess the quality of all of the included studies. In the Jadad scale, 4–7 stars indicate high-quality studies, while 0–3 stars indicate low-quality studies. In the NOS, 7, 5–7, and 0–4 stars indicate high-, moderate-, and low-quality studies, respectively.

### Statistical analysis

Effect size combinations were analyzed using Stata/SE 15.0 (StatCorp, College Station, TX, USA). The *I*^2^ statistic was used to describe the degree of heterogeneity between the studies. *I*^2^ > 50% is considered to indicate high heterogeneity, in which case a random effects model is recommended. Meanwhile, when *I*^2^ < 50%, a fixed effects model is typically used for analysis. Pooled relative risk and odds ratio (*OR*) values and 95% confidence interval (95%*CI*) estimates were calculated using Stata/SE 15.0, to determine the relationship between post-immune infection and the risk of severe disease in COVID-19 patients. Potential publication bias was evaluated through the visual inspection of funnel plots, and Egger’s test was used to assess the symmetry of the funnel plots. The level of statistical significance was defined as *P* < 0.05.

## Results

### Literature retrieval and quality evaluation

**Fig. 1:**
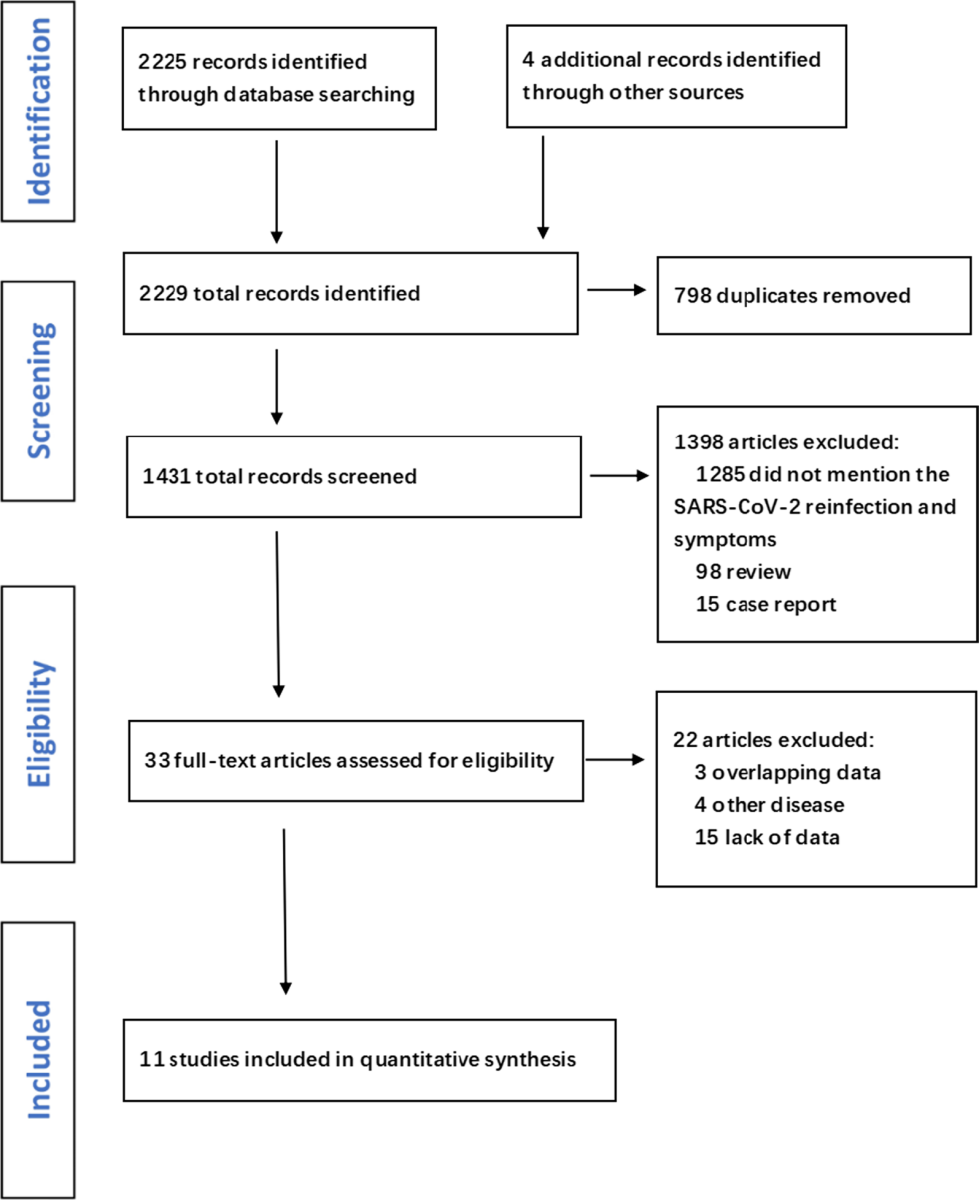
Flow chart of literature search and screening

A search of recently published literature in the databases identified 2229 records. Of these, 2196 were excluded during the screening phase (title and abstract review) and the remaining 33 were fully apprised. Based on the inclusion and exclusion criteria, 11 studies [11–21] were finally included in the meta-analysis (Fig. 1), and the details of these studies are provided in Table 1. The meta-analysis included 211,413 subjects, with 1132 reinfected patients. The mean time to reinfection ranged from 50.5 days to 201 days in the six observational studies. Two of the papers included in the review were published in 2020 and nine in 2021. Six of these studies used an observational design and five were randomized controlled trials. The studies were mostly conducted in European countries, such as England, and the United States. Women comprised a large proportion of the study population. The quality of all of the included studies was evaluated using the Jadad scale or the NOS.

**Table 1:** Characteristics of the included studies

Study author, year	Country	Study type	Median age	Age range	Male(%,case/control)	Severe/immune	Severe/naive	Mild/immune	Mild/naive	NOS/Jadad
Adrielle Dos Santos Letícia et al. (2021)[23]	Brazil	Observational study	39.2	18–58	21.2/37.1	12	35	21	27	5
Hall et al. (2021)[12]	England	Observational study	45.7	18–85	17.2/14.9	50	1126	105	578	6
Murillo-Zamora et al. (2021)[13]	Mexico	Observational study	NA	≥ 20	NA	38	48,057	220	84,131	5
Bailly et al. (2021)[14]	France	Observational study	87.0	≥ 77	NA	2	4	11	1	5
Singh et al. (2021)[15]	India	Observational study	55.0	50–63	35.6/64.4	89	211	98	179	8
Slezak et al. (2021)[16]	America	Observational study	NA	≥ 15	NA	29	4094	286	71,055	6
Baden et al. (2020)[17]	America	RCT	51.4	≥ 18	52.2/53.1	4	43	7	142	6
Logunov et al. (2021)[18]	Russia	RCT	NA	≥ 18	61.1/61.5	0	20	16	42	8
Polack et al. (2020)[19]	Multinational	RCT	52	16–91	51.1/50.1	1	9	7	153	7
Sadoff et al. (2021)[20]	Multinational	RCT	52	≥ 18	55.1/54.7	14	60	103	291	7
Madhi et al. (2021)[21]	South Africa	RCT	NA	18–65	NA	4	6	15	17	6

*NA* Not available, *NOS* Newcastle–Ottawa scale, *Jadad* Modified Jadad scale

Severe/immune: Cases diagnosed as severe after natural infection/vaccination

Severe/naive: Cases diagnosed as severe in the initial infection

Mild/immune: Cases diagnosed as mild after natural infection/vaccination

Mild/naive: Cases diagnosed as mild in the initial infection

### Association between post-immune SARS-CoV-2 infection and disease severity

**Fig. 2:**
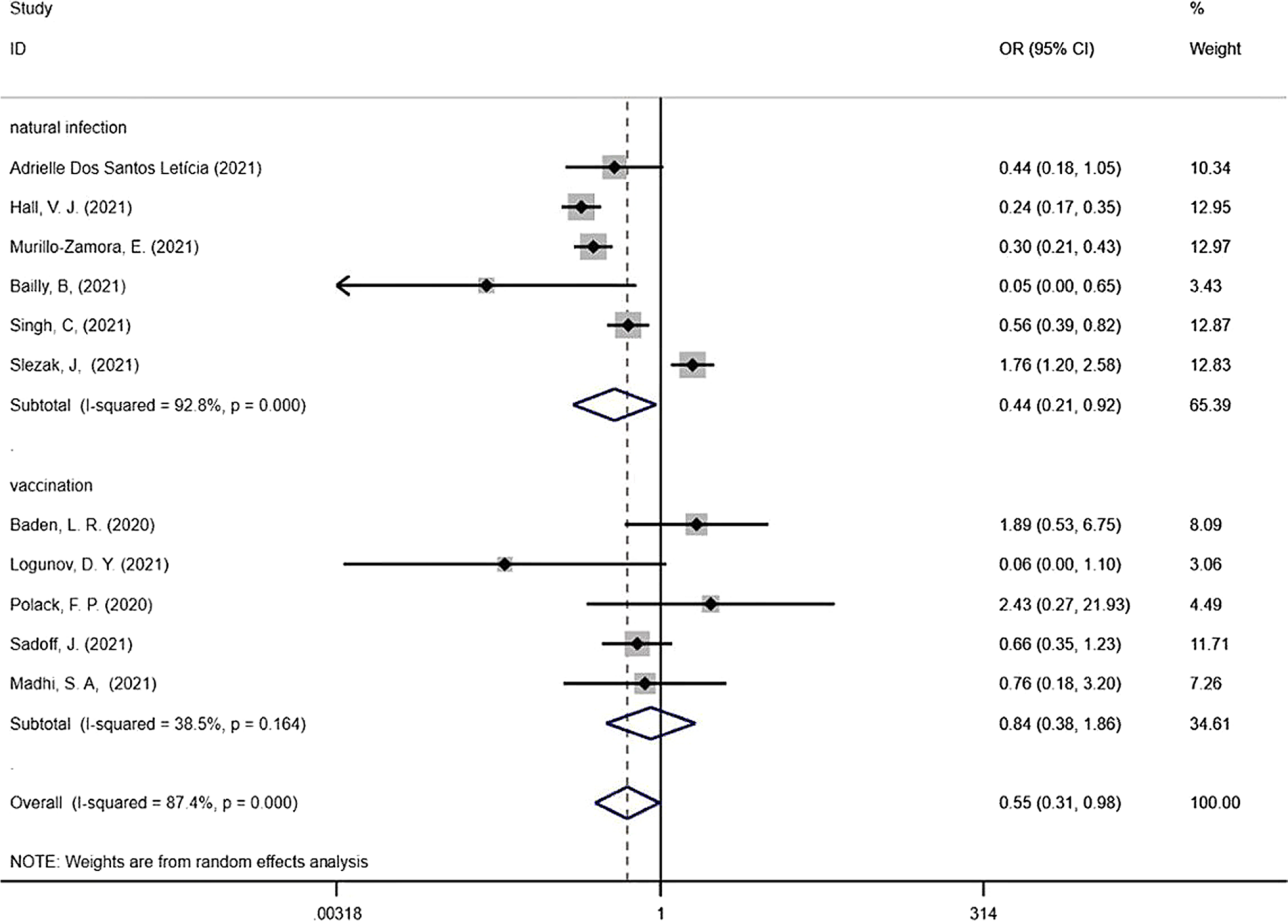
Forest plots showing pooled risk ratio of SARS-CoV-2 post-immune infection associated with severe cases (the dashed line represents the combined *OR* value)

The correlation between post-immune SARS-CoV-2 infection and disease severity was analysed using a random effects model, based on the degree of heterogeneity of the included studies (*I*^2^ > 50%). The *OR* of severe symptoms in reinfected COVID-19 patients was calculated. The case group included confirmed severe cases, and the control group included mild cases. The pooled results indicated that previous infection or vaccination was a protective factor against severe COVID-19 (*OR* = 0.55, 95%*CI* = 0.31–0.98). A subgroup analysis was then performed based on the type of post-immune infection (Fig. 2), i.e., reinfection after initial infection and breakthrough infection after vaccination. The random effects model gave an *OR* of 0.44 (95%*CI* = 0.21–0.92) for reinfection compared with an initial infection and the fixed effects model gave an *OR* of 0.84 (95%*CI* = 0.38–1.86) for breakthrough infection after vaccination compared with an initial infection. These results indicated that the immune protection provided by an initial infection or vaccination protects against severe disease upon post-immune infection.

### Publication bias and sensitivity analyses

**Fig. 3:**
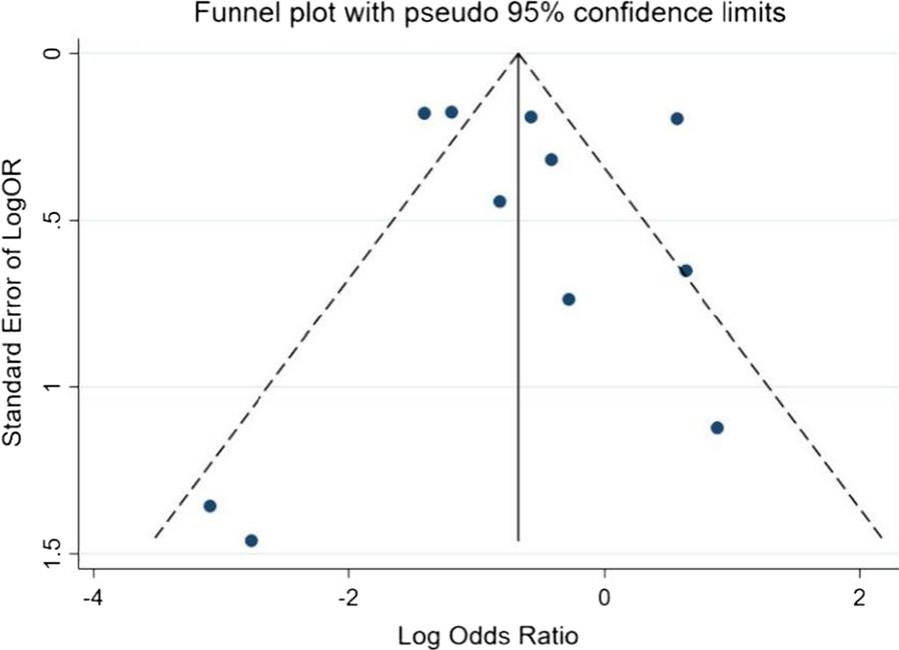
Funnel plot for evaluation of publication bias

**Fig. 4:**
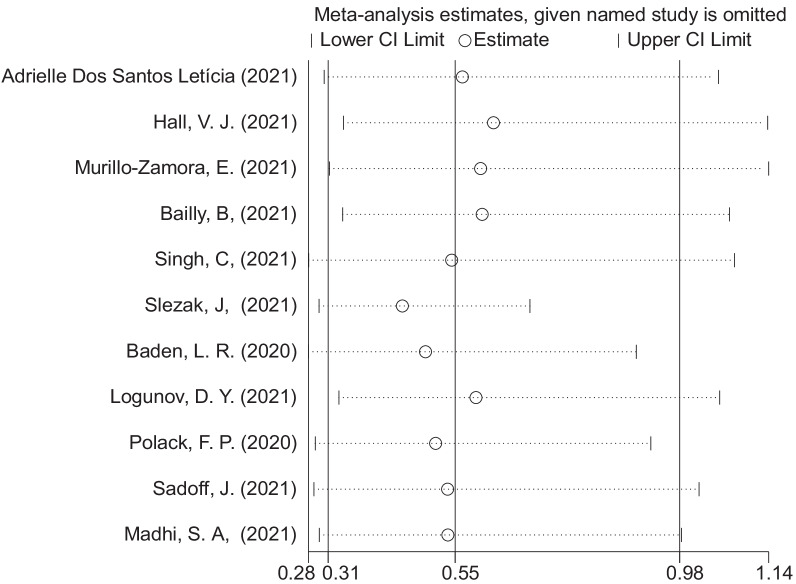
Sensitivity analysis

Potential publication bias was assessed using funnel plots and Egger’s regression test. No evidence of publication bias was found (Fig. 3, Egger’s test: *P* = 0.859). Sensitivity analysis showed that the exclusion of some studies had no remarkable effect on the results (Fig. 4).

## Discussion

SARS-CoV-2 has spread globally, creating the COVID-19 pandemic, which has posed a significant threat to public health and the global economy. Vaccination is one of the most effective public health interventions during a pandemic. Currently available SARS-CoV-2 vaccines are safe for most people, including those with pre-existing conditions of any type, including auto-immune disorders. SARS-CoV-2 vaccination program has been implemented in more than 200 countries, and more than half of the world’s population has received at least one dose of a SARS-CoV-2 vaccine [22–24].

Vaccines work by training and preparing the body’s natural defences provided by the immune system to recognise and eradicate the viruses they target. After vaccination, if the body is later exposed to the virus, it is immediately ready to destroy it, so that illness is prevented [20, 25–27]. A major concern during the COVID-19 pandemic is that protective immunity may be transient. A previous study showed that generating protective immunity against SARS-CoV-2 is possible in humans, either following a natural infection or after inoculation with a vaccine [28]. A recently published study in Qatar showed that reinfections had a 90% lower risk of resulting in serious disease than primary infections. In accordance with the results of the current meta-analysis, initial infection and vaccination were found to be protective factors against severe COVID-19 symptoms upon post-immune infection, indicating that both primary SARS-CoV-2 infection and vaccination provide adequate protection against severe clinical outcomes.

One potential hurdle for SARS-CoV-2 vaccination acceptance is the risk of ADE, which may increase the severity of COVID-19. Considering earlier reports of the effect of ADE on SARS-CoV and MERS-CoV infections, the hypothesis that ADE may occur with SARS-CoV-2 infection has existed since the outbreak of COVID-19 [29–32]. To date, numerous in vivo and in vitro experiments have been conducted to explore the occurrence of ADE in patients infected with SARS-CoV-2. However, only one in vitro study has preliminarily demonstrated the possibility of ADE in SARS-CoV-2 infections [9]. Moreover, there is no evidence that ADE occurs in the SARS-CoV-2-infected population. Thus, we cannot definitively determine whether ADE occurs in the reinfected population at present. More population studies of SARS-CoV-2 must be conducted to clarify the role of ADE. Some people are unwilling to be vaccinated because of concerns about the effectiveness and safety of the vaccines [33–36]. The findings of this review may alleviate vaccine hesitancy to a certain extent, and a change in the attitude of some individuals may increase COVID-19 vaccine acceptance rates.

Our study is the first meta-analysis of the association between SARS-CoV-2 post-immune infection and disease severity. Identifying reinfection requires detecting the virus at two different time points and using viral genomic data to distinguish reinfection from persistent viral carriage. The major limitation of the current meta-analysis is that most studies of post-immune infection have not addressed the issue of virus variants during reinfection or the initial infection or the specific vaccine administered. However, the number of vaccine doses, the type of the vaccination, and the SARS-CoV-2 strain may be associated with ADE and may play a role in post-immune infection and disease severity. However, due to the limited amount of data, we were unable to perform an in-depth analysis of these potential associations. More studies are needed to explore the relationship between these factors and ADE. The second limitation is that we were not able to compare the age and sex of severe cases and mild cases, as these data were not reported in the published articles. Finally, the data included in this meta-analysis were published before 25 October 2021, and at that time the Omicron strain of SARS-CoV-2 had not emerged. Thus, data about reinfections and breakthrough infections with the Omicron strain were not included. However, a research report showed that the symptoms of breakthrough infection with the Omicron strain are mild, which is consistent with the results of this review [37]. Another study showed that a breakthrough infection with the Omicron variant was equivalent to a vaccine booster at enhancing the protective effect of the vaccine [38]. These results further demonstrate the lack of ADE in COVID-19 patients. Despite its limitations, this study demonstrated that natural infection with or vaccination against SARS-CoV-2 alleviates the severe outcomes of post-immune infection and that ADE is not likely to occur. This finding may help increase the public’s confidence in SARS-CoV-2 vaccines. Approved SARS-CoV-2 vaccines provide a high degree of protection against serious illness and death from the disease, but no vaccine is 100% protective [39, 40]. Vaccines also prevent serious illness even after contracting SARS-CoV-2 infection [41]. Our findings may provide guidance for vaccination programmes, promote global vaccination, and help end the COVID-19 epidemic.

## Conclusions

In conclusion, the results of our meta-analysis showed that natural infection with or vaccination against SARS-CoV-2 are protective factors against severe symptoms upon post-immune infection. However, further research is needed to confirm the conclusion that ADE does not occur with SARS-CoV-2 infection.

## Data Availability

All relevant data supporting the findings is contained within the manuscript. Database used for statistical calculations is available on request (contact Lin Gan, one of the authors of this article).
